# Acute stress disorder in patients with traumatic spinal cord injury: risk factors and coping strategies

**DOI:** 10.3389/fpsyt.2025.1555589

**Published:** 2025-05-19

**Authors:** Haohua Shi, Yufang Su, Chunyan Pan

**Affiliations:** Department of Orthopedic, The First Affiliated Hospital of Soochow University, Suzhou, Jiangsu, China

**Keywords:** acute stress disorder, traumatic spinal cord injury, treatment, care, nursing

## Abstract

**Background:**

Traumatic Spinal Cord Injury (TSCI) exerts a profound negative impact on patients’ psychological well-being and daily life. The objective of this study is to dissect the comorbidity of Acute Stress Disorder (ASD) among TSCI patients, identify its contributing factors, and construct a predictive model to provide empirical support for clinical treatment and nursing care.

**Methods:**

Patients with TSCI admitted to our hospital between January 2022 and September 2024 were enrolled in this study. We collected and compared general demographic and disease-related data between patients who developed ASD and those who did not.

**Results:**

A cohort of 224 individuals with TSCI was enrolled in the study, revealing an incidence rate of ASD to be 33.93%. Age (r=0.562), gender (r=0.489), monthly household income (r=0.585), and injury severity (r=0.722) were correlated with ASD. Age ≤45 years (OR=2.606, 95%CI: 1.985-3.215), female gender (OR=2.213, 95%CI: 2.004-2.612), monthly household income less than 5000 Renminbi (RMB) (OR=3.027, 95%CI: 2.677-3.431), and level A injury severity (OR=3.673, 95%CI: 3.115-4.066) were the independent predictors of ASD among patients with TSCI. The area under the ROC curve (AUC) and its 95% confidence interval (CI) were 0.788 (0.710, 0.852) for the predication model, indicating good sensitivity and specificity.

**Conclusion:**

ASD is a common occurrence in patients with TSCI, with a multitude of contributing factors. The predictive model established in this study aids in the risk assessment of ASD in patients with TSCI.

## Introduction

Spinal cord injury is a severe neurological condition that exerts profound effects on the physical, psychological, and vocational aspects of an individual’s life (1). The global prevalence of spinal cord injury is estimated to range between 20 to 30 million people, with approximately 900,000 new cases emerging annually (2). In China, the current population of individuals living with spinal cord injury is estimated to be around 3.74 million, with an annual increase of about 90,000 new cases (3). This condition predominantly affects younger individuals, with a marked male predominance, and exhibits significant regional variations in incidence rates (4). Common etiologies of spinal cord injury include traffic accidents, falls from heights, and acts of violence (5). Spinal cord injury resulting from these traumatic events is characterized by high incidence and mortality rates, abrupt onset, and complexity, along with inherent uncertainties (6). Individuals with spinal cord injury often contend with a multitude of complications, which can have devastating impacts on their physical and mental health, impose substantial financial burdens, and potentially trigger psychological stress responses (7). Consequently, early intervention in the form of treatment and care is of paramount importance for improving the prognosis of spinal cord injury patients (8).

Acute Stress Disorder (ASD) is a constellation of stress response symptoms that manifest within 2 to 28 days following exposure to a traumatic event (9). If left undetected and untreated, ASD may progress to Post-traumatic Stress Disorder (PTSD). Among trauma patients, both ASD and PTSD are not only prevalent but also exert a profound impact on the physical and psychological well-being of individuals (10, 11). While PTSD has garnered significant attention from researchers worldwide due to its potential to cause irreversible psychiatric impairments, research on ASD in the context of Traumatic Spinal Cord Injury (TSCI) patients remains relatively scarce, with the current state of occurrence and contributing factors requiring further elucidation. Consequently, the aim of this study is to investigate the risk factors for ASD in TSCI patients and to develop a predictive model that delineates the associated risk factors, to provide a scientific foundation for early psychological intervention and nursing care in TSCI patients.

## Methods

This study utilized a retrospective design, and the research protocol was formally approved by the hospital’s Ethics Committee (approval number: 2025394). All participating patients had signed the written informed consents.

This study enrolled patients with TSCI admitted to our hospital from January 2022 to September 2024. The specific inclusion criteria were as follows: firstly, patients had to be diagnosed with spinal cord injury through magnetic resonance imaging (MRI) or computed tomography (CT) scans; secondly, patients were required to be over the age of 18 and have a clear history of trauma; thirdly, the time from TSCI injury to enrollment had to be within 28 days; and lastly, both the patients and their families had to provide informed consent to participate in the study. In terms of exclusion criteria, this study excluded patients with spinal cord injuries caused by non-traumatic events, those who experienced additional stressors within 28 days, and those who declined to participate in the study. These stringent inclusion and exclusion criteria were applied to ensure the homogeneity of the study population and the reliability of the research findings.

In the present study, we employed the Stanford Acute Stress Reaction Questionnaire (SASRQ) to evaluate the presence and severity of Acute Stress Disorder (ASD) among patients. The SASRQ has been extensively reported and validated in prior research (12, 13). This self-report questionnaire consists of 30 symptom items, which are distributed across five dimensions: acute dissociative symptoms (10 items), re-experiencing of the traumatic event (6 items), avoidance reactions (6 items), irritability symptoms (6 items), and social dysfunction (2 items). Higher scores on the SASRQ indicate more severe symptoms. The scale has demonstrated robust psychometric properties, with a Cronbach’s α coefficient of 0.913, confirming its reliability and validity (14). The total score of the SASRQ ranges from 0 to 150, with a score of 40 or above indicating the presence of ASD. Based on this criterion, patients were divided into two groups: the ASD group and the non-ASD group. This classification facilitated a comparative analysis between individuals who developed ASD symptoms and those who did not, thereby providing a structured and methodologically sound approach to assess the impact and characteristics of ASD within our study population.

We extracted the following characteristics of the patients from medical records and nursing documentation: age, gender, Body Mass Index (BMI), marital status, educational level, monthly household income, reasons for traumatic spinal cord injury, injury segment, and injury severity. This comprehensive data collection is essential for a analysis of the factors associated with ASD in the context of TSCI.

### Quality control

To ensure the accuracy and consistency of the data presented in this study, we implemented a series of rigorous quality control measures throughout the research process. All data were collected using standardized protocols and instruments, with researchers receiving comprehensive training to minimize variability and ensure uniformity in data recording. Data entry was performed by two independent researchers to reduce the risk of transcription errors, with discrepancies resolved through cross-checking and verification against the original data sources. Regular monitoring and auditing procedures were conducted to assess data quality, including periodic reviews of data collection forms, validation of entered data against source documents, and checks for completeness and consistency. A comprehensive data cleaning process was also implemented to identify and correct any inconsistencies or outliers through statistical checks for data plausibility and logical consistency, as well as manual review of flagged data points. Detailed records of all data collection, entry, and validation processes were meticulously maintained to ensure full transparency and traceability of data handling procedures. Through these comprehensive quality control measures, we aimed to ensure that the data presented in this study are accurate, reliable, and consistent, thereby supporting the validity and robustness of our findings.

In this study, statistical analysis was conducted using SPSS 25.0 software. Quantitative data that were normally distributed are presented as the mean ± standard deviation, while quantitative data that did not follow a normal distribution are depicted using the median and interquartile range (M(Q1,Q3)). For between-group comparisons, independent samples t-tests were employed for normally distributed quantitative data, and Mann-Whitney U tests were used for non-normally distributed quantitative data. Qualitative data are described in terms of frequency and percentage composition, with between-group comparisons made using chi-square tests and Fisher’s exact probability method. Ordinal data were analyzed using rank sum tests. All variables that demonstrated statistical significance were included in a logistic regression analysis to explore the influencing factors of ASD. Subsequently, this study developed a risk prediction model for ASD in patients with TSCI. To assess the goodness-of-fit of the model, the Hosmer-Lemeshow (H-L) test was conducted. Additionally, the sensitivity and specificity of the risk model were determined based on the area under the receiver operating characteristic (ROC) curve. This comprehensive evaluation ensures the model’s predictive accuracy and its clinical utility in identifying patients at risk for ASD following TSCI. The study utilized two-tailed tests with a significance level set at 0.05.

## Results

A total of 224 patients with TSCI were enrolled in this study, with 76 of these patients diagnosed with ASD, resulting in an incidence rate of ASD among TSCI patients of 33.93%. As delineated in **Table 1**, significant differences were observed in age, gender, monthly household income, and injury severity between the ASD and non-ASD groups (all P < 0.05).

**Table 1:** The characteristics of patients with traumatic spinal cord injury.

Characteristics	Acute stress disorder group (n=76)	No acute stress disorder group(n=148)	t/χ^2^	P
Age(y)	42.10 ± 6.36	48.31 ± 7.44	6.242	0.010
Male/female	48/28	123/25	1.007	0.001
BMI (kg/m^2^)	24.22 ± 3.08	24.31 ± 2.95	4.326	0.086
Marital status			1.455	0.103
Married	61(80.26%)	119(80.41%)		
Unmarried	15(19.74%)	29(19.59%)		
Educational level			1.214	0.116
Elementary education	2(2.63%)	4(2.70%)		
High school education	14(18.42%)	33(22.30%)		
Associate degree	36(47.37%)	71(47.97%)		
Bachelor’s degree	22(28.95%)	37(25.00%)		
Graduate education	2(2.63%)	3(2.03%)		
Monthly household income (RMB)			1.670	0.024
<5000	52(68.42%)	31(20.95%)		
5000~10000	16(21.05%)	88(59.46%)		
>10000	8(10.53%)	29(19.59%)		
Reasons for traumatic spinal cord injury			1.839	0.115
Falls	39(51.32%)	81(54.73%)		
Traffic accidents	29(38.16%)	55(37.16%)		
Crush injuries	8(10.52%)	12(8.11%)		
Injury segment			1.335	0.102
Cervical segment	28(36.84%)	50(33.78%)		
Thoracic segment	34(44.74%)	68(45.95%)		
Lumbar segment	13(17.11%)	29(19.59%)		
Sacral segment	1(1.32%)	1(0.68%)		
Injury severity			1.866	0.004
Level A	45(59.21%)	51(34.46%)		
Level B	14(18.43%)	30(20.27%)		
Level C	11(14.47%)	36(24.32%)		
Level D	6(7.89%)	31(20.95%)		

BMI, body mass index; y, years; RMB, Renminbi.

As presented in **Table 2**, the correlation analysis revealed significant correlations between several variables and the occurrence of ASD among TSCI patients. Specifically, age (r=0.562), gender (r=0.489), monthly household income (r=0.585), and injury severity (r=0.722) all demonstrated statistically significant correlations with the development of ASD (all P < 0.05).

**Table 2:** Correlation analysis on the acute stress disorder and characteristics of patients with traumatic spinal cord injury.

Variables	r	P
Age(y)	0.562	0.017
Gender	0.489	0.041
BMI (kg/m^2^)	0.144	0.096
Marital status	0.183	0.125
Educational level	0.210	0.073
Monthly household income (RMB)	0.585	0.036
Reasons for traumatic spinal cord injury	0.148	0.097
Injury segment	0.167	0.102
Injury severity	0.722	0.001

BMI, body mass index; y, years; RMB, Renminbi.

In this study, variables that exhibited statistical significance in the univariate analysis were selected for further multivariate logistic regression analysis. The allocation of variables for the multivariate logistic regression analysis is detailed in **Table 3**. As depicted in **Table 4**, the findings from the logistic regression analysis identified age ≤ 45 years (OR=2.606, 95%CI: 1.985-3.215), female gender (OR=2.213, 95%CI: 2.004-2.612), monthly household income less than 5000 RMB (OR=3.027, 95%CI: 2.677-3.431), and level A injury severity (OR=3.673, 95%CI: 3.115-4.066) as the independent predictors of ASD among patients with TSCI (all P < 0.05).

**Table 3:** The variable assignment for multivariate logistic regression analysis on the occurrence of acute stress disorder in patients with traumatic spinal cord injury.

Factors	Variables	Assignment
Acute stress disorder	Y	Yes=1, no=2
Age(y)	X_1_	≤45 = 1, >45 = 2
Gender	X_2_	Female=1, male=2
Monthly household income (RMB)	X_3_	<5000 = 1, >5000 = 2
Injury severity	X_4_	Level A =1, level B =2, level C =3, level D =4

y, years; RMB, Renminbi.

**Table 4:** Logistic regression analysis on the influencing factors of acute stress disorder in patients with traumatic spinal cord injury.

Variables	β	Wald	OR	95%CI	P
Age ≤45y	0.190	0.115	2.606	1.985-3.215	0.019
Female	0.154	0.103	2.213	2.004-2.612	0.034
Monthly household income<5000 RMB	0.128	0.111	3.027	2.677-3.431	0.016
Level A injury	0.174	0.120	3.673	3.115-4.066	0.002

y, years; RMB, Renminbi.

**Figure 1:**
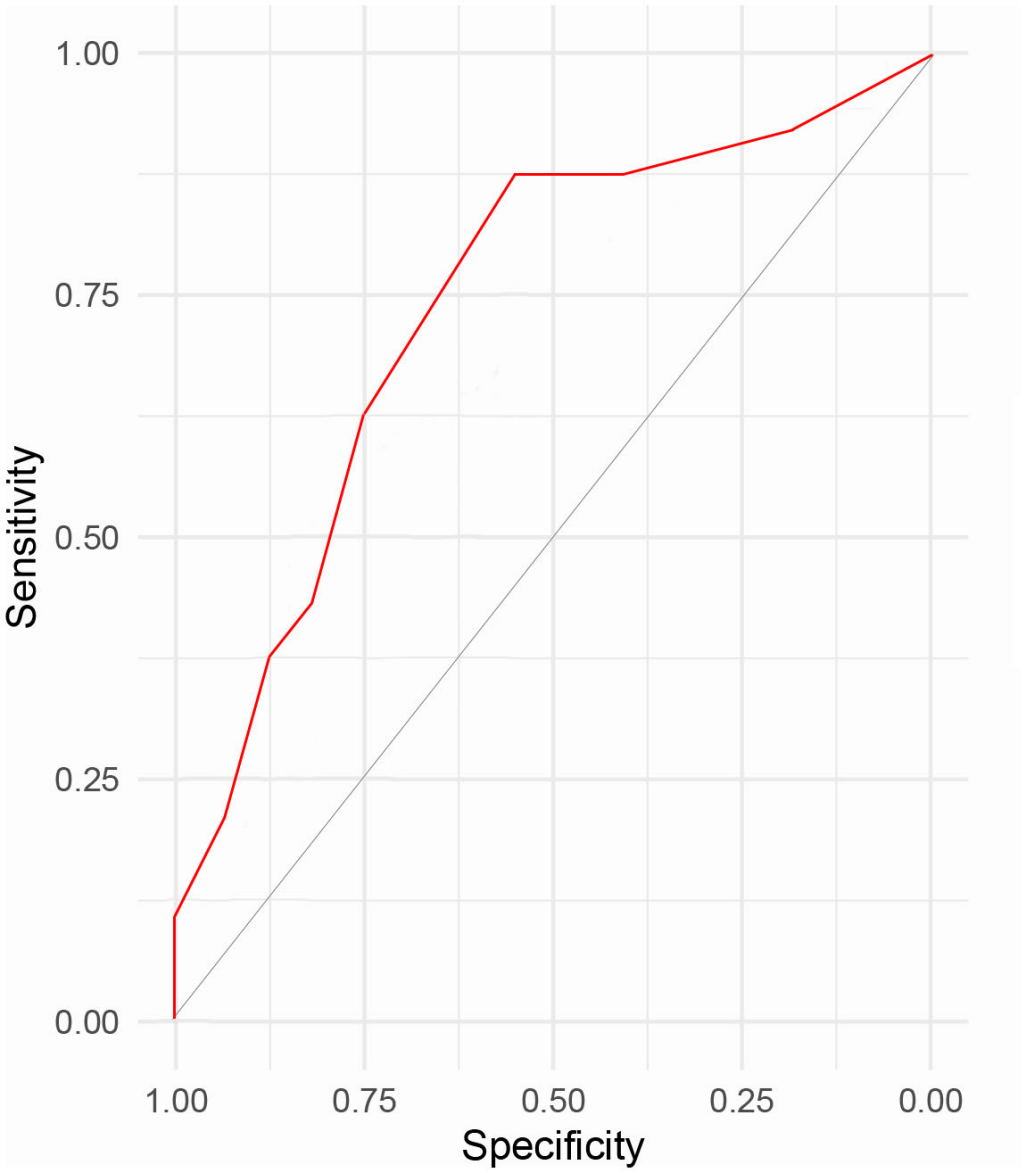
The ROC curve of the risk prediction model for acute stress disorder in patients with traumatic spinal cord injury.

As detailed in **Table 5**, this study established the scoring criteria for a predictive model of ASD among patients with TSCI. Utilizing the ROC curve depicted in **Figure 1** and the scoring criteria of the risk prediction model, we calculated and compared the sensitivity and specificity of the prediction model across various cut-off scores, and consequently derived the Youden index (sensitivity + specificity - 1) for each. **Table 6** reveals that the Youden index was optimized when the total score range was between 5.5 to 6.5. Consequently, a total score of 6 was determined as the cut-off value for this risk prediction model. At a total score of 6, the prediction model demonstrated high sensitivity and specificity. The area under the ROC curve (AUC) and its 95% confidence interval (CI) were 0.788 (0.710, 0.852), signifying that the risk prediction model possessed robust discriminative power to identify the occurrence of ASD among TSCI patients (all P < 0.05). These findings underscore the model’s effectiveness in clinical settings for predicting ASD risk.

**Table 5:** The scoring method of the logistic model for the risk of acute stress disorder in patients with traumatic spinal cord injury.

Variable	Score
Age ≤45y	2
Female	2
Monthly household income<5000 RMB	3
Level A injury	3

y, years; RMB, Renminbi.

**Table 6:** The sensitivity and specificity of the prediction model of acute stress disorder in patients with traumatic spinal cord injury under different cuff values.

Total score	Sensitivity	Specificity	Youden index
-1.0	1.000	0.000	0.000
1.0	1.000	0.124	0.124
1.5	0.973	0.236	0.209
2.5	0.920	0.383	0.303
3.5	0.859	0.551	0.410
4.5	0.821	0.699	0.520
5.5	0.681	0.860	0.541
6.5	0.677	0.888	0.565
7.5	0.241	0.908	0.149
8.5	0.134	0.926	0.060
9.5	0.106	0.939	0.045
10.5	0.029	1.000	0.029
11	0.000	1.000	0.000

## Discussions

For patients with TSCI, the transition from a healthy state to one characterized by paralysis with potential bowel, bladder, or sexual dysfunction leads to drastic changes in lifestyle. Research indicates that psychological factors such as self-stigma, burden (including loss of social function and financial strain), and low self-esteem severely impede social interactions for these patients (15, 16). Particularly among younger populations, the psychological impact of this adaptive challenge can be devastating (17). These factors may serve as stressors that precipitate ASD. ASD, being one of the earliest psychological issues to emerge post-injury, necessitates early identification of patients at risk for stress-related disorders to minimize the incidence of psychological impairments (18, 19). Furthermore, studies (20, 21) suggest that TSCI patients are at a higher risk of developing new-onset anxiety or depression after discharge compared to patients with other conditions, especially those under the age of 50. This underscores the importance of early psychological intervention for TSCI patients. The findings of this study reveal that the incidence of ASD among TSCI patients is 33.9%, with the occurrence of ASD being significantly associated with patient age, gender, monthly household income, and injury severity. These correlations underscore the necessity for clinical healthcare providers to implement early interventions based on these influential factors in order to mitigate the onset of ASD.

Younger patients with TSCI demonstrate a significantly higher likelihood of developing ASD subsequent to traumatic events. This observation is consistent with the broader literature (22) on ASD, which highlights the increased vulnerability of younger individuals to stress-related psychopathologies. The heightened susceptibility of younger TSCI patients to ASD can be attributed to the ongoing neurodevelopmental processes during adolescence and early adulthood. During this period, the brain is still undergoing significant maturation, particularly in regions associated with emotional regulation and stress response. Consequently, exposure to trauma and stress may have more pronounced and enduring neurodevelopmental consequences in younger individuals compared to their older counterparts. A study on children aged 6 to 12 (23) has revealed a higher prevalence of ASD in males, suggesting that gender may be a risk factor for ASD. Given the male predominance in the incidence of TSCI, this gender-based risk factor may also be relevant to TSCI patients. To address this potential vulnerability, intervention strategies emphasize the critical need for early identification and intervention in ASD (24). Specifically, a two-tiered screening process and multidisciplinary collaboration are advocated to ensure timely detection and comprehensive management of ASD. For TSCI patients, this approach could involve integrating mental health assessments into post-injury care protocols. Additionally, care coordination models such as the Shared Plan of Care (SPoC), which are designed to provide comprehensive, family-centered care for individuals with ASD (25), could be adapted for younger TSCI patients.

Gender appears to significantly influence the risk of developing ASD among patients with TSCI, with female patients exhibiting a higher risk compared to their male counterparts. This observation aligns with findings from relevant studies (26, 27). The gender disparity may be attributed to several factors, including differences in brain physiological structure and activity patterns between men and women, as well as the potential influence of lower estrogen levels in females. Lower estrogen levels may predispose women to more persistent fearful memories in the context of stressors (28). In this study, the primary stressor leading to spinal cord injury in females was traffic accidents, predominantly affecting middle-aged and younger individuals. These women often face substantial psychological stress due to pressures from work, life, and marital issues. However, the complexity of gender differences in ASD and the ongoing debate surrounding these findings highlight the need for additional studies to better understand the potential impact of gender on ASD prevalence and symptomatology in TSCI patients. In clinical nursing practice, healthcare providers may pay special attention to female patients. Regular psychological assessments are necessary for female TSCI patients to promptly identify psychological issues and to offer tailored psychological support and counseling (29, 30).

Economic income is a significant factor influencing the occurrence of ASD in patients with TSCI. The TSCI patient demographic is predominantly middle-aged and young adults who are the primary earners in their households, bearing the responsibility of caring for elderly family members and children, and shouldering substantial familial and societal duties (31, 32). The onset of TSCI not only severely impacts the individual’s health and quality of life but also places a tremendous financial strain on their families. This increased economic pressure, compounded by the potential loss of the patient’s ability to work, exacerbates the family’s financial burden and, consequently, the risk of ASD in the patient (33). When managing TSCI patients, it is crucial to consider the impact of economic factors on their psychological health. Research (34) indicates that financial burden is a critical determinant of mental health among individuals with chronic conditions, especially in developing countries like China, where the economic burden of chronic diseases is on the rise and outpaces the growth of Gross Domestic Product (GDP). For TSCI patients, this financial burden can be particularly onerous, given that their treatment and rehabilitation may require long-term and costly medical interventions (35). Therefore, the care plan for TSCI patients should encompass not only medical and rehabilitative support but also economic and social assistance. This may involve providing patients with financial counseling, information on social welfare benefits, and employment assistance to alleviate their financial stress as part of a comprehensive care plan.

The severity of injury grading is a significant factor influencing the occurrence of ASD in TSCI patients. This phenomenon may be associated with the more complex treatment regimens required for patients with severe conditions, the increased severity of paralysis, and the potential life-threatening nature of their injuries. Consequently, these patients may necessitate extended stays in the Intensive Care Unit (ICU), which can exacerbate psychological stress responses. The ICU environment itself poses a unique stressor for patients, potentially leading to a range of physiological injuries such as stress ulcers, stress hyperglycemia, and stress hypertension (36, 37). Psychological stress is a complex process involving multiple factors from physiological, psychological, and socioenvironmental domains. These factors are communicated systemically through the neuroendocrine-immune network, potentially causing damage to the function of various systems and organs (38). Therefore, for TSCI patients with higher injury grades, healthcare providers must pay increased attention to their psychological state, promptly recognizing and intervening in psychological stress responses. This includes offering psychological support, pain management, sleep interventions, and pharmacological treatments when necessary (39). Additionally, healthcare providers should work closely with patients and their families, providing essential information and resources to help them cope with financial and emotional stress, as well as the challenges posed by prolonged hospitalization and potential functional impairments (40).

This study has identified the potential risk factors for ASD in patients with TSCI and has developed a predictive model to assess the risk of ASD development in this patient population. The high sensitivity of this model renders it a potent tool for healthcare providers to identify early ASD risks, devise targeted intervention strategies, and reduce the incidence of ASD. These findings underscore the necessity of conducting psychological assessments in clinical nursing practice for TSCI patients to promptly recognize their psychological needs and offer timely support. Research outcomes indicate that emotional, financial, and psychological support from family members, healthcare professionals, and various social support groups play a significant role in mitigating the occurrence of post-traumatic psychological stress (41, 42). This comprehensive support provides individuals who have experienced trauma with essential resources, enabling them to better manage the psychological pressures associated with trauma and thereby reducing the risk of ASD development.

This study has made certain achievements in exploring the risk factors for ASD in TSCI patients and in constructing predictive models; however, there are several limitations that warrant further consideration. Firstly, as a single-center study with a relatively small sample size, there is a potential for selection bias in terms of population or geographic distribution, which may restrict the generalizability of the findings. Secondly, the retrospective design of this study lacks systematic follow-up of the subjects, necessitating future prospective studies to further validate the predictive efficacy of the model in the development of ASD. Thirdly, the study only addresses the occurrence of ASD within 28 days post-TSCI, and the long-term incidence of ASD and its contributing factors require additional research for supplementation and refinement. Future research should delve into the trajectory of psychological stress responses in TSCI patients with the aim of refining risk prediction models and continuously refining and optimizing them in clinical practice.

## Conclusion

Inconclusion, this study unveiled the high comorbidity of ASD among TSCI patients and has identified age, gender, monthly household income, and injury severity as influential factors in the occurrence of ASD. The ASD risk assessment model developed for TSCI patients within this research indicates that patients with a score of 6 or above are at a higher risk of developing ASD. This study not only provides a scientific foundation for evaluating the risk of ASD in TSCI patients but also offers guiding recommendations for the implementation of psychological intervention measures. By constructing and applying this predictive model, healthcare professionals may more effectively identify high-risk patient groups and take targeted preventive actions. The findings of this study underscore the importance of early identification and intervention, providing scientific support for personalized care in clinical practice, which is conducive to enhancing the quality of care and psychological health levels of TSCI patients.

## Data Availability

The raw data supporting the conclusions of this article will be made available by the authors, without undue reservation.
